# Engaging people who use drugs in policy and program development: A review of the literature

**DOI:** 10.1186/1747-597X-7-47

**Published:** 2012-11-24

**Authors:** Lianping Ti, Despina Tzemis, Jane A Buxton

**Affiliations:** 1 British Columbia Centre for Excellence in HIV/AIDS, St. Paul’s Hospital, 608-1081 Burrard Street, Vancouver, BC, V6Z 1Y6, Canada; 2 School of Population and Public Health, University of British Columbia, 2206 East Mall, Vancouver, BC, V6T 1Z3, Canada; 3 British Columbia Centre for Disease Control, 655 West 12th Avenue, Vancouver, BC, V5Z 4R4, Canada

**Keywords:** People who use drugs, Peer engagement, Policy development, Program development

## Abstract

Health policies and programs are increasingly being driven by people from the community to more effectively address their needs. While a large body of evidence supports peer engagement in the context of policy and program development for various populations, little is known about this form of engagement among people who use drugs (PWUD). Therefore, a narrative literature review was undertaken to provide an overview of this topic. Searches of PubMed and Academic Search Premier databases covering 1995–2010 were conducted to identify articles assessing peer engagement in policy and program development. In total, 19 articles were included for review. Our findings indicate that PWUD face many challenges that restrict their ability to engage with public health professionals and policy makers, including the high levels of stigma and discrimination that persist among this population. Although the literature shows that many international organizations are recommending the involvement of PWUD in policy and program development, our findings revealed a lack of published data on the implementation of these efforts. Gaps in the current evidence highlight the need for additional research to explore and document the engagement of PWUD in the areas of policy and program development. Further, efforts to minimize stigmatizing barriers associated with illicit drug use are urgently needed to improve the engagement of PWUD in decision making processes.

## Introduction

On a global scale, community-based methods have been increasingly used as an effective public health approach to engage various populations in addressing concerns about their health [1,2]. Evidence supporting the engagement of people with lived experience or ‘peers’ at different stages of policy, program and research development shows positive health outcomes for populations [3-5]. In order for decision makers to improve the health of individuals and make services more relevant to the target population, policies and practices must be based on the needs of that population. Allowing the voices of peers to be heard is crucial for developing a deeper understanding of complex health problems. By doing so, initiatives to tackle these health issues will have a greater impact on the target population by improving the acceptability and utilization of programs for these individuals and by extension, increase accessibility to these services [6].

Despite the growing interest of involving peers in areas of decision making, there is currently no established definition of the terms ‘peers‘ and ‘peer engagement’. Depending on the literature, peer engagement can differ in varying degrees. A continuum of peer engagement can range anywhere between tokenism, where peers have limited influence in the decision making process and are only consulted to create a false appearance of inclusiveness, to full, collaborative involvement, where peers are involved at a more active level and in all stages of policy and program development [7]. Taken from a number of articles, the term ‘peers’ in this literature review will refer to any persons of equal standing within a particular community who share a common lived experience [8,9]. A ‘community’ is a group of individuals living in a particular area or place. For example, in Vancouver, Canada, people living in the downtown eastside (DTES) are referred to as the DTES community, and people who use drugs (PWUD) living in the DTES are referred to as peers among other PWUD. Peer engagement is a community-based approach and we have defined it as the process of consulting and collaborating with decision makers using a bottom-up approach in order to better address the needs of the community. Additionally, we have defined the terms policy and program development as the following: ‘policy development’ is the process of forming guiding principles and rules for improving the health of populations, whereas ‘program development’ is the process of developing projects or services that aim to improve the health of populations.

While a large body of evidence supports the use of peer-based services and interventions, little is known about the success of peer engagement at an earlier, more influential stage in the decision making process. This literature review aims to provide a summary of the available evidence on peer engagement among PWUD and its role in policy and program development. Findings from this review will identify gaps in the literature as well as provide important information on how to more effectively engage peers in policy and program making decisions.

## Methods

During June and July 2011, the search for literature material was conducted using PubMed and Academic Search Premier databases and spans the period 1995 to 2010. Using Medical Subject Heading terms and Boolean terms to identify papers that dealt with the topic of peer engagement in policy and program development, database searches were performed multiple times with one or more combinations of the following terms: peer, peer-based, experiential worker, HIV, injection drug users, policy development, community, participation, user involvement, drug policy, partnership. These search terms were identified from the authors’ prior knowledge and preliminary searches about this particular topic. A cited reference search and a grey literature search were also conducted. As well, back referencing from included studies was performed to search for additional relevant articles. Most of the grey literature was found through one main search engine, Google, and included literature such as government and United Nations reports, conference papers, and discussion papers.

The studies that were selected for review were limited to articles published in English and were those that discussed peer engagement in relation to policy and program development decision making. Articles that did not meet these criteria were excluded from the review. Participatory action research (PAR) and community-based participatory research (CBPR) are two community-involved research methods that are often included in literature searches related to peers. However, PAR and CBPR articles that did not discuss any peer engagement in policy or program settings were excluded from the review. Additionally, given that our focus was specific to PWUD populations, we excluded all articles related to other populations from the literature review. We noted that the majority of literature in our search pertained to hospital patients and youth populations, in comparison to the population of interest in this study.

Titles and abstracts of all articles were briefly reviewed and potentially relevant articles were saved for further examination. The saved articles were then entirely read through and only the documents that mentioned peer engagement with PWUD at the policy and program development stage were included in the review. Once all the articles for inclusion were identified, a content analysis was conducted. After a second read, emerging themes were noted and quantified as they appeared in the text. Connections between and within these themes and categories were then explored.

## Findings

**Figure 1:**
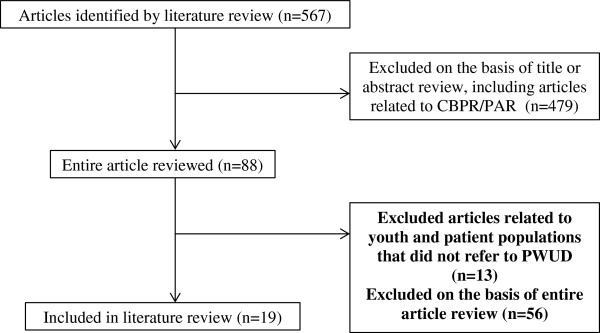
**Flow diagram for literature review on peer engagement in policy and program development, 1995-2010. **CBPR: community-based participatory research, PAR: participatory action research, PWUD: people who use drugs.

As indicated in Figure 1, searches of electronic databases, grey literature, and searches of reference lists of included studies yielded 567 articles and documents in total. Of the 567 references, 88 had the potential to be relevant based on their titles and abstracts. 69 of these references were excluded after reading the entire article. In total, 19 articles and documents were included and contributed to the findings. Table 1 lists the literature used to conduct the present literature review.

**Table 1:** Documents included in the literature review

**Author**	**Year**	**Study type**	**Country**	**Aims**	**Results/outcomes**
*Challenges and barriers involving highly stigmatized populations in policy making decisions*					
Bryant et al.	2008	Qualitative research	Australia	To describe beliefs about consumer participation in drug treatment services and perceived barriers to consumer participation	The majority of consumers and providers believed in consumer participation; barriers to consumer participation included opinions that it is not the consumers' place to take part, and the lack of interest and skills to participate
Fischer & Neale	2008	Qualitative research	Scotland, England	To explore challenges in the involvement of illicit drug users in the decisions about their treatment	Challenges to involving drug users in treatment decisions include lack of financial resources, communication between professionals and clients, and lack of investment in education, training and skills
Halloran et al.	1996	Pilot project evaluation	USA	To report on the development, implementation and evaluation of Project LEAP, a psychoeducational intervention, to increase participation of PLWHA	Participation from organizations increased from an average of 0.5 organizations at baseline to 2.3 at follow-up; increase in self-esteem, self-confidence, and knowledge were seen in the organizations
Roy & Cain	2001	Participatory action research	Canada	To examine the barriers and obstacles to meaningful involvement of PLWHA	Stereotyping of PLWHA, fear of losing anonymity, usefulness of PLWHA, power imbalances, health concerns are among the barriers that limit the involvement of PLWHA in the development of policies and delivery of services
Travers et al.	2008	Cross-sectional	Canada	To examine barriers and facilitating factors to the greater involvement of PLWHA in community-based research	Challenges to involving PLWHA include HIV-related stigma, health-related challenges, mistrust, and credibility of PLWHA; facilitating factors include training opportunities, financial compensation, building trust
*Success in programs and interventions involving PWUD*					
Booth et al.	2009	Intervention study	Ukraine	To investigate changes in needle- related risks among IDU following peer leader interventions	Peer leaders significantly reduced needle risk behaviors at 6 months follow-up compared to baseline; findings suggest that peer leader intervention approach may be effective in reducing HIV risk behaviors among IDU in Ukraine
Broadhead et al.	1998	Intervention study	USA	To compare the TOI with a PDI in the context of HIV prevention efforts	Both interventions significantly reduced HIV risk behaviors; PDI reached a larger and more ethnically/geographically diverse population of IDU at a lower expense than TOI
Canadian Public Health Association	2005	Report	Canada	To lay out the ideal response to HIV/AIDS in Canada	Encourages sharing of responsibilities and increasing partnerships to make more effective use of our knowledge, skills and resources
Crofts & Herkt	1995	Literature review	Australia	To review the literature on the histories and impact of user groups in Australia	The role of user groups in Australia is important to the government for preventing further transmission of HIV among IDU and engagement with the groups should continue
Garfein et al.	2007	Randomized control trial	USA	To investigate whether a peer-education intervention can reduce injection and sexual risk behaviors associated with HIV and Hepatitis C in young IDU	The peer intervention reduced injection risk behaviors among young IDU by 29% overall at 6 months postintervention compared to control and 76% reduction compared to baseline; Sexual risk behaviors were also decreased postintervention
Hayashi et al.	2010	Cohort study	Canada	To evaluate a peer-run outreach-based syringe exchange program by VANDU called the Alley Patrol	The Alley Patrol was successful in reaching a higher risk group of IDU and was significantly associated with lower levels of needle reuse (AOR=0.65)
Latkin et al.	2003	Intervention study	USA	To investigate whether a network-oriented peer outreach intervention is associated with HIV prevention among drug users	In the experimental group, participants were 3 times more likely to report reduction in injection risk behavior and 4 times more likely to report increased condom use than controls; peer outreach strategies may be useful in reducing HIV risk behaviors in drug using communities
Purcell et al.	2007	Intervention study	USA	To investigate the efficacy of a peerHIV prevention program with PWUD through a project called the Risk Avoidance Partnership* project	Participants reported significant reductions of injection and sexual risk behaviors compared to baseline but there were no significant changes in medical outcomes
Weeks et al.	2009	Intervention study	USA	To investigate outcomes of a peer HIV prevention program with PWUD through a project called "The Risk Avoidance Partnership" project	Results show a significant HIV risk reduction among all study participants at 6 months follow-up compared to baseline
*A call for increasing the engagement of PWUD in policy making decisions*					
Canadian HIV/AIDS Legal Network	2005	Report	Canada	To explain why PWUD need to be involved in the response to blood borne diseases and drug use	Recommendations to increase meaningful involvement of PWUD were highlighted
Charlois	2009	Report	Netherlands	To address issues of substance use and trafficking at frontline levels	Recommendations to increase involvement of drug users' participation through expertise and practice sharing
Kerr et al.	2006	Community-based case study	Canada	To document the activities and structure of VANDU using a community-based case methodology	VANDU is highly involved in advocacy, activism, and public education of PWUD and provide support and care for their peers
Osborn & Small	2006	Response article	Canada	To understand the role of PWUD in influencing drug policies in Vancouver, BC	Organizations such as VANDU have enormous impact on municipal drug policy through activism
UNAIDS	2007	Policy brief	-	To highlight challenges, actions, and the importance of the greater involvement of PLWHA	Recommendations to achieving greater involvement of PLWHA through government actions and actions from organizations of PLWHA; challenges include weak management, low skill levels, lack of funding

PWUD: people who use drugs.

IDU: people who inject drugs.

PWLHA: people living with HIV/AIDS.

AOR: adjusted odds ratio.

VANDU: Vancouver Area Network of Drug Users.

TOI: traditional outreach intervention.

PDI: peer driven intervention.

A review of the literature identified a number of themes related to peer engagement in policy and program development in various settings. The themes that emerged were quantified and categorized broadly and consisted of the following: 1) challenges and barriers to involving highly stigmatized populations in policy making decisions; 2) success in programs and interventions involving PWUD; and 3) a call for increasing the engagement of PWUD in policy and program decisions.

### Challenges and barriers to involving highly stigmatized populations in decision making

Stigma has previously been defined as “deeply discrediting” and reduces the bearer “from a whole and usual person to a tainted, discounted one” [10]. The literature indicates that people who are part of a stigmatized population, such as PWUD and people living with HIV/AIDS (PLWHA), face numerous challenges and obstacles regarding their involvement in policy and program development. Barriers such as the criminalization, stigmatization and discrimination of drug use have prevented many PWUD from getting involved in health policy and program planning [11,12]. As an example, Fischer and Neale (2008) noted that clients found that “many healthcare professionals implicitly or explicitly blamed drug users for their addiction, treated them with contempt, or disregarded many of their problems. Such negative attitudes often impeded participation of PWUD in treatment decision making by undermining their self-worth and self-confidence [12].” Such perceptions can hinder engagement with PWUD in policy and program planning. In some settings, structural factors (i.e., funding, capacity) may limit the ability of organizations to involve PWUD in their planning agendas. As well, power imbalances between PWUD and professionals have also contributed to the difficulties of engaging with this population [12,13].

Furthermore, there have been difficulties involving PLWHA in many health organizations. Some of these barriers include having to disclose their HIV status [14] as well as persistent negative attitudes towards PLWHA [15]. Similar to PWUD, many agencies and professionals reserve power over these stigmatized individuals, limiting their involvement in making decisions and planning services that directly impact their health [15,16]. As a result of the obstacles that peers face, overcoming these barriers are crucial to involving highly stigmatized peers in policy and program development.

### Success in programs and interventions involving PWUD

Peer-run services and interventions have resulted in positive health outcomes for many PWUD [17,18]. In addition to reaching a more diverse population of people who inject drugs (IDU), Broadhead et al. (1998) also concluded that peer-driven interventions reduced HIV-related risk behaviors among IDU. A range of HIV risk behaviors, including a decrease in sharing injecting paraphernalia and frequency of injecting among IDU was noted from peer outreach and peer-driven interventions [1]. Two other articles indicated that PWUD who were exposed to peer outreach were more likely than controls who were not exposed to peer outreach to report both a reduction in risk behaviors related to injection and an increase in the use of condoms [19,20]. In addition to the success of peer interventions on reducing risk behaviors among IDU, two articles reported a reduction in injection risk behavior among young IDU who received training to become peer outreach workers [3,21]. Additionally, Garfein et al. (2007) showed fewer instances of unprotected sex among participants of a peer education intervention at six months follow-up compared to the control baseline [3].

There have been a few countries that have made advances and gained success in engaging peers in policy and program development, including Canada and Australia. Canada has seen a rise in the involvement of PWUD in response to the HIV/AIDS epidemic [11]. An increasing proportion of PWUD have been invited to participate in policy meetings and other action plans, such as the HIV/AIDS action plan, *Leading Together: Canada Takes Action on HIV/AIDS*[22]. Likewise in Australia, IDU are active and maintain a dominant role in the decisions around policy and program development in relation to harm reduction [23]. However, Crofts and Herkt (1995) argue that although there are IDU and IDU groups that have been extensively involved in the decision making process around their health, their contributions have been poorly documented in literature [23]. The reasons for this include the everyday challenges that PWUD face and are described in the section above.

### A call for implementation: increasing the involvement of PWUD in policy making

Many documents have stressed the importance of this type of peer-based approach in policy development. The Canadian HIV/AIDS Legal Network, in the document “Nothing About Us Without Us”, recommends that:

“People who use drugs need to be meaningfully involved in consultative processes, as well as in decision-making or policy-making bodies and advisory structures dealing with issues related to HIV/AIDS, HCV, and illegal drugs… In practice, people who use drugs should be invited to participate in all consultations, committees, or fora where policies, interventions, or services concerning them are planned, discussed, researched, determined, or evaluated” [11].

In the 6^th^ EXASS Net meeting in Amsterdam, the Netherlands also concluded that there is value in collaborating with PWUD to develop better and more effective drug policies and strategies [24]. Formed by the Pompidou Group of the Council of Europe, EXASS Net is a European network of frontline workers in the health, social, and law enforcement sectors that aim to address issues of substance use and trafficking at frontline levels. On another scale, UNAIDS (2007) also advocates for increasing public participation and meaningful inclusion of PLWHA in policy and program development as well as in the implementation of these programs. The policy brief highlights that the greater involvement of PLWHA will increase the effectiveness, acceptability, and usability of HIV-related policies and programs [25]. Other research studies have also promoted the engagement of peers in the decision making process. For example, an article by Kerr et al. (2006) discusses the benefits of incorporating the activities of peer-run organizations such as the Vancouver Area Network of Drug Users (VANDU) into frameworks for policy and program development [26]. VANDU is a grassroots organization of current and former PWUD who work to provide peer-based education and support for PWUD as well as advocates for changes in public policy and practice. VANDU representatives are being increasingly involved in policy meetings, including in the national AIDS strategy [26]. Another article that highlights the important work of VANDU encourages PWUD to be more actively involved in policy making [27]. Osborn and Small (2006) note that the alleviation and increased effectiveness of the problems around drug use calls for PWUD to “be centrally involved in deciding and implementing the response to the problem” [27]. These articles highlight the value of involving and collaborating with peers and peer-run organizations and calls for similar work to document the benefits of this peer-based type of approach.

## Discussion

This review identified many countries that strongly encourage the involvement of peers at strategic levels in policy and program decisions, including Canada, United Kingdom (UK), United States, and the Netherlands. While a large body of scientific evidence have reported positive outcomes from peer-run programs and interventions for PWUD, such as the reduction of risk behaviors and frequency of injecting [1,19], less attention has been paid to peers and their involvement at more upstream levels in policy and program development.

PWUD are important stakeholders in the issues surrounding substance use and health, yet there is limited documentation on their collaborative efforts with policy makers and program planners. This review highlighted the challenges and obstacles that prevent peers from becoming more engaged in decision making processes. Barriers of stigma and discrimination may have made it more difficult for policy makers to appreciate the benefits of involving peers in policy decisions [11,12]. In order to improve and develop practices around this issue, future efforts should first focus on actively reducing issues of social stigmatization. Free from barriers, peers can more effectively engage in policy and program development.

There have been minimal examples in the available literature of strongly identified peer groups advocating for the health and well being of their peers. In Canada, VANDU is well known for their dominant voice in the matters of policy agendas. Based on a social movement model, VANDU’s democratic grassroots approach is continuously challenging public policies, and shifting social attitudes and awareness towards PWUD. Recently, VANDU protesters were responsible for shutting down a street in Vancouver in a rally for stable housing opportunities [28]. Another example of their involvement in the community includes their ongoing support of Insite, Vancouver’s first officially sanctioned supervised injection facility. Further, VANDU has succeeded in engaging peers in various practices by identifying and implementing interventions that are needed to reduce harms associated with drug use, including working with: the British Columbia (BC) harm reduction program to provide naloxone (Narcan^©^) to opioid users, Vancouver Coastal Health Authority for safer smoking education initiatives, and with the University of BC researchers for education and support meetings for people who drink illicit alcohol [29-31]. However, in other settings, peer groups have not played roles that represent the same level of involvement as VANDU. A major challenge that many peer groups face worldwide in running their organizations are the limited resources available and lack of funding from the government [23,32]. Without adequate funding, peer groups remain unstable and are ineffective as advocators. Therefore, governments should increase their efforts in financially supporting peer groups as well as to encourage and assist the formation of new peer groups in various settings. Additionally, governments should develop a system where institutional boundaries do not limit the participation of this population (e.g. research writing skills to write a competitive grant proposal, requirement to be affiliated with a university).

In addition to grassroots activities, various governments globally have been making advancements in engaging with peers in policy formation and development. For example, the BC Ministry of Health developed a model called ‘Patients as Partners’ to highlight the importance of equal representation and collaboration between all stakeholders affected by the same issue [33]. The BC harm reduction program in Canada follows these guiding principles by including PWUD from across BC in policy decisions and program changes in their efforts to improve the health of the population [34,35]. Furthermore, in 2010, the BC Ministry of Health Services launched the ‘Healthy Minds, Health People’ initiative, which is a ten-year plan that calls for collective action between public and private sector stakeholders, as well as community partners to promote positive health in BC [36]. In the UK, the Substance Misuse Service User Involvement Project commissioned by the Wandsworth Care Alliance facilitates the engagement of PWUD and alcohol in revising policy and delivery of treatment services to the population [37]. Collectively, these efforts highlight the progress countries are making to acknowledge the valuable contribution that PWUD can make to policy.

There are several areas of policy and program development that without the insight of PWUD these issues may not have been identified and/or programs would not be effective [38]. These include but may not be limited to: policies around supportive housing and supportive assistance, decriminalizing drug use, informing appropriate drug paraphernalia needed for safer drug use, increasing access to naloxone, informing best practices for harm reduction and addiction treatment including opioid maintenance therapy, and health promotion initiatives such as effective messaging for overdose prevention and response, as well as relevant educational materials. There may, however, be challenges in engaging with peers in policy and program development particularly when disagreements between peers and professionals in clinical decision making (e.g., opioid substitution therapy dose levels, supervised dispensing of medication) may hinder the ability to make appropriate decisions. Efforts to ensure checks in the balance of power between professionals and peers are crucial in these situations.

Evaluation of the contributions made by PWUD can be conducted through documenting policy changes over time and monitoring the effectiveness of programs. Depending on the policy or program, this evaluation may be conducted in the short, intermediate, or long term. As discussed previously, there is a need to publish findings from these evaluations in order to inform policy and program developers of the value of engaging with PWUD in the decisions around their lives. The engagement of PWUD can be further assessed using a tool such as Hart’s Ladder of Participation [39] or using a process evaluation tool whereby PWUD are asked to describe their experiences being engaged in policy and program development.

As highlighted here, there have been many examples of the successes of engaging with PWUD in the areas of policy and program development. Unfortunately, these examples were not published in a way that was identified in this narrative review. This may imply that others searching for evidence regarding the effectiveness of including PWUD in policy and program development may not have found these examples either. Therefore, efforts should be made to publish alternate versions of non-academic literature within publicly indexed academic journals. In addition, increasing the referencing of non-academic material within academic articles may be effective in incorporating non-academic articles within the searchable literature. Regardless, the engagement of PWUD has greatly influenced governments’ approaches to addressing the needs of this community. For example, in BC, a recent peer evaluation project on harm reduction drug paraphernalia identified the need for more relevant supplies to be distributed in order to address the changing drug use trends in the area [34]. Additionally, peers have also been involved in informing their own health services needs. For example, peers identified the messaging of a recent coroner’s alert on heroin overdose to be inappropriate despite their efforts to warn PWUD about the “potent” and “strong” heroin circulating in the area. Instead, this message encouraged PWUD to seek out this drug and thereby, increase their risk of overdose [35].

This literature review demonstrates the lack of published data available on the initiatives taken by health professionals to include peers in policy and program discussions and meetings. We found this to be under representative of the work being done in this area. Although the overall literature on the subject does in fact incorporate significant references to peer involvement in research using PAR and CBPR methodologies, such articles were excluded from the review as their focus lies more on research processes and less on how these processes can actually contribute to policy and program development, which is the key theme of this review. This may also be a reflection of the research interest of academic journals themselves or that peer-run organizations may not have the expertise in academic writing to submit to peer review journals. The reliance on peer engagement in these approaches supports the need for further research to explore connections between PAR, CBPR and policy and program development in order to determine whether these types of research methods can be translated into policy making decisions by peers. Increased efforts are needed to provide evidence-based materials in order to make progress in this area.

We should note that the literature search process revealed a large body of literature on patient and youth populations, which we excluded from the review as it did not meet our inclusion criteria. Nevertheless, these articles point to the importance of engaging with peers in making decisions that directly affect them [4,40,41]. Within the healthcare sector, the importance of patient engagement has been increasingly recognized as an effective approach for public health interventions [41]. These efforts to engage with patients and youth have been implemented in many countries, including the Netherlands and the UK [41-43]. Given that involvement at the policy development stage has shown high success and effectiveness in patient and youth populations, the authors’ argue that this success can also be transitioned over to other populations such as PWUD.

Despite the objective approach taken in this literature review, several limitations present themselves. First, this is not an exhaustive illustration of peer engagement in the context of policy and program development. The method used to conduct this review and the selection criteria may have limited the results of the literature review. The lack of published literature may be due to the fact that this topic may not necessarily have been published in the searchable peer-reviewed literature. In addition, our search in the grey literature may not have captured all documentation of engaging with peers. Hence, this analysis may not be reflective of all the work currently being done in this area among PWUD. Second, there may be a publication bias, given that significant findings are more likely to be published than inconclusive results.

## Conclusion

The literature review identified a consistent knowledge gap in the subject of peer engagement in relation to policy and program development and highlights how far behind we currently are in developing such initiatives with PWUD. With clearly effective outcomes for various populations, efforts to minimize stigmatizing attitudes towards PWUD among health and service professionals and policy makers may assist in improving peer collaboration in policy agendas. Moreover, future research should seek to further explore and document peer engagement in the context of policy and program development. Additionally, we recommend that individuals already involved in this area continue to publish their findings in peer-reviewed journals and elsewhere. Peers have an ethical and imperative right to be involved in the decisions affecting their lives and often, they are the ones who are the most knowledgeable on how to most effectively approach their population [11]. A decision to involve peers in the policies and practices around their health will not only give strength to their voices but our collaborative efforts will more effectively address the needs of this community.

## Competing interests

The authors declare that they have no competing interests.

## Authors’ contributions

The specific contributions of each author is as follows: LT and JB were responsible for study design; LT was responsible for the collection of the literature and prepared the first draft of the analysis; All authors provided critical comments on the first draft of the manuscript and approved the final version to be submitted.
